# Detection of non-pathogenic and pathogenic populations of *Vibrio parahaemolyticus* in various samples by the conventional, quantitative and droplet digital PCRs

**DOI:** 10.1038/s41598-024-54753-y

**Published:** 2024-02-19

**Authors:** Sinisa Vidovic, Roland Taylor, Duncan Hedderley, Graham C. Fletcher, Nicola Wei

**Affiliations:** 1 The New Zealand Institute for Plant and Food Research Limited, 120 Mount Albert Road, Sandringham, 1025 Auckland, New Zealand; 2 The New Zealand Institute for Plant and Food Research Limited, Palmerston North, New Zealand; 3 McGill University, Montreal, Canada

**Keywords:** *Vibrio parahaemolyticus*, Emerging pathogen, Vibriosis, Diagnostics, Seafood safety, Biological techniques, Microbiology

## Abstract

In this study, three generations of polymerase chain reaction (PCR) assays: (*i*) conventional PCR, (*ii*) qPCR and (*iii*) droplet digital PCR (ddPCR), were systematically tested for their abilities to detect non-pathogenic and pathogenic populations of *Vibrio parahaemolyticus*. The limit of detection (LOD) for the ddPCR was 1.1 pg/µL of purified DNA, followed by the qPCR (5.6 pg/µL) and the conventional PCR (8.8 pg/µL). Regarding the LOD for *V. parahaemolyticus* cells, the ddPCR assay was able to detect 29 cells, followed by the conventional PCR assay (58 cells) and the qPCR assay (115 cells). Regarding the sensitivities to detect this pathogen from PCR inhibition prone samples (naturally contaminated mussels), the ddPCR assay significantly outperformed the conventional PCR and qPCR. The ddPCR assay was able to consistently detect non-pathogenic and pathogenic populations of *V. parahaemolyticus* from naturally contaminated mussels, indicating its tolerance to various PCR inhibitors. This study also revealed the significant difference between conventional PCR and qPCR. The conventional PCR assay showed significantly greater sensitivity than that of the qPCR assay in detecting *V. parahaemolyticus* in crude samples, whereas the qPCR assay showed better sensitivity in detecting the presence of *V. parahaemolyticus* in purified DNA samples.

## Introduction

*Vibrio parahaemolyticus,* a Gram-negative halophilic bacterium, is ubiquitously present in estuarine marine and coastal environments. Although many strains of *V. parahaemolyticus* can be harmless to humans, certain pathogenic strains of this bacterial species can cause gastroenteritis, septicaemia, and wound infections^1^. Indeed, *V. parahaemolyticus* is the leading cause of gastrointestinal infections associated with the consumption of seafood worldwide^2–4^. Vibriosis caused by *V. parahaemolyticus* is mainly associated with symptoms such as fever, headache, vomiting, abdominal cramps and diarrhoea. The gastroenteritis-type infection is self-limiting^5^. *V. parahaemolyticus* outbreaks are mainly associated with the consumption of molluscan shellfish, a concern for public health and the seafood industry in many parts of the world^6–8^. Not only have the frequencies of these outbreaks increased in the last two decades^9^, but also outbreaks associated with *V. parahaemolyticus* have been recorded in geographic regions that previously never experienced such outbreaks^10^. New Zealand has experienced outbreaks of vibriosis in winter months^11^, when concentrations of *V. parahaemolyticus* in shellfish are invariably low^7^. Therefore, the employment of sensitive and reliable diagnostic methods for the detection of the total population of *V. parahaemolyticus* and its more pathogenic sub-population^12–16^ plays an important role in the prevention of this food-borne infectious disease.

Two major virulence factors, thermostable direct hemolysin (TDH) and thermostable-related hemolysin (TRH), are commonly associated with the clinical isolates^17^. Specifically, TDH, which produces ß-type haemolysis on Wagatsuma agar, is commonly associated with the population of clinical *V. parahaemolyticus* isolates, but it is less commonly present among environmental isolates^18,19^, confirming its importance in the pathogenicity of *V. parahaemolyticus*. In addition to these two haemolysins, *V*. *parahaemolyticus* possess two type III secretion systems, T3SS1 and T3SS2, responsible for invasion of the host cells and survival of the pathogen within the host cell^20^. In general, T3SS1 is commonly present in *V. parahaemolyticus* isolates and plays a role in autophagy^21^ and cytotoxicity^22^. In contrast, T3SS2 is rarely found among populations of *V. parahaemolyticus,* and it is suspected that this secretion system plays a role in enterotoxicity^22^.

Reliable diagnostic data are crucial for development of effective measures to prevent outbreaks associated with this organism. Therefore, we carried out a study to validate three molecular assays for the detection of *V. parahaemolyticus* in different sample matrix. These diagnostic assays are based on three different PCR platforms, including conventional PCR, quantitative PCR (qPCR), and droplet digital PCR (ddPCR). The PCR-based assays were simultaneously tested for the detection of species and pathogen-specific markers of *V*. *parahaemolyticus*. The assays were tested using three laboratory-based approaches: (*i*) genomic DNA, (*ii*) viable cells of *V. parahaemolyticus,* and (*iii*) spiked muscle tissue. Besides the laboratory-based approaches, all PCR assays were tested against 480 freshly harvested mussels for the presence of *V. parahaemolyticus*.

## Material and methods

### Strains of *V. parahaemolyticus*

Two strains of *V. parahaemolyticus* of clinical origin were used for the laboratory-based studies. The first strain, 19ER2200 (*tdh* and *trh* positive), was obtained from the Institute of Environmental Science and Research (ESR), New Zealand. This strain was the causative agent of the first *V. parahaemolyticus* outbreak in New Zealand, in June 2019. The second strain, F11-3A (*tdh* and *trh* positive) isolated in Washington state, USA, was associated with a 1997 US outbreak. Both strains belong to the pandemic sequence type (ST)36. *Pseudomonas fluorescens* strain cc848406E was used as a negative control. The isolates were stored at – 80 °C in Luria–Bertani (LB) broth (Difco) with 10% glycerol. For each laboratory-based experiment, fresh cultures derived from the frozen stocks were used.

### Diagnostic PCR assays

Three multiplex PCR platforms, a conventional multiplex ST36 PCR^14^, a multiplex qPCR^15^, and a multiplex ddPCR^16^, were tested for their sensitivities to detect *V. parahaemolyticus*. Oligonucleotide primers and probes used in this study are presented in Table 1.

**Table 1:** Oligonucleotide primers used for the PCR assays.

Gene	Type of PCR assay	Forward primer sequence (5′–3′)	Reverse primer sequence (5′–3′)	Reference
*tlh*	ST36 PCR	AGAACTTCATCTTGATGACACTGC	GCTACTTTCTAGCATTTTCTCTGC	^14^
*tdh*		GTAAAGGTCTCTGACTTTTGGAC	TGGAATAGAACCTTCATCTTCACC	^31^
*trh*		CATAACAAACATATGCCCATTTCCG	TTGGCTTCGATATTTTCAGTATCT	^31^
*prp*		CGGCTTGAGTTTTCGTCATT	CCACACCTGCTGGTTATTTAGTTC	^14^
*flp*		TGGTTGTGTTTAGAGCAGGG	TGTTGGTAATACGATAAGAATGAGA	^14^
*tlh*	qPCR	ACTCAACACAAGAAGAGAT CGACAA	GATGAGCGGTTGATGTCCAA	^15^
*tlh* probe		CGCTCGCGTTCACGAAA CCGT		^15^
*trh*		TTGCTTTCAGTTTGCTATTGGCT	TGTTTACCGTCATATAGGCGCTT	^15^
*trh* probe		AGAAATACAACAATCAAAACTGA		^15^
*tdh*		TCCCTTTTCCTGCCCCC	CGCTGCCATTGTATAGTCTTTATC	^15^
*tdh* probe		TGACATCCTACATGACTGTG		^15^
*tlh*	ddPCR	GAACGCAGACATTACG	ACCACTTTGTTGATTTGA	^16^
*tlh* probe		CATTGCTGCGTCGTTGCTCC		^16^
*tdh*		GGTCAGGAAGTTCGT	ACGGCATAGGTGAGTA	^16^
*tdh* probe		CCGCCACGACAGTTACGA		^16^
*ureR*		GCACTCTAACACCCAA	AGCTGATACATCGGTT	^16^
*ureR* probe		CTAGGCGAGCAAAAGCACTCT		^16^

Conventional and ddPCR were carried out using the T100 and C1000 Touch^™^ thermal cyclers (Bio-Rad, USA), respectively while the qPCR assay was carried out using the 7500 Fast Real-Time PCR System (Applied Biosystems). Droplets for ddPCR were generated with the automated droplet generator (Bio-Rad, USA). The PCR reactions were carried out in a 20 µL reaction mixture using 2 µL of PCR template, as previously described^14,15^. Only the ddPCR assay was slightly modified. Briefly, the PCR reaction mixture (20 µL) contained 2 × ddPCR Supermix for Probes (no dUTP) (Bio-Rad, USA), 990 nM of all three pair of primers, and 250 nM of *ureR* (FAM 1), 125 nM of *tlh* (FAM 2), as well as 500 nM of *tdh* (HEX) probes. The samples were treated for 10 min at 95 °C, following 39 cycles at 94 °C for 30 s and 56 °C for 60 s and one cycle at 98° C for 10 min. The digital droplets were detected by the QX 200^™^ Droplet Reader (Bio-Rad, USA) and analysed by QX Manager software, standard edition, version 2.0 (Bio-Rad, USA). Each laboratory-based experiment was carried out on three different occasions (biological replications) using two technical replications for each sample.

### Extraction and preparation of genomic DNA

*Vibrio parahaemolyticus* strains were cultured from frozen stocks on Tryptone Soy Agar (TSA) plates (Difco) supplemented with 2% NaCl followed by overnight incubation at 35 °C. Three single colonies were picked by a sterile cotton swab and resuspended in 1 mL of 0.9% saline. After centrifugation at 10,000×*g* for 5 min, the supernatant was removed and genomic DNA was extracted using a Qiagen DNeasy Tissue kit (Qiagen Inc., Valencia, CA) according to the manufacturer’s instructions. After this, the genomic DNA from each ST36 strain, F11-3A and 19ER2200, was equally combined (v/v), followed by serial dilutions using fivefold dilution steps. The first three concentrations of DNA were determined with a NanoDrop^™^ microvolume spectrophotometer, whereas the rest of the dilutions were calculated since these DNA concentrations went beyond the sensitivity limit of the NanoDrop instrument. The DNA concentration of each PCR template used in this experiment is indicated in Table 2.

**Table 2:** Concentration of *Vibrio parahaemolyticus* DNA samples used for PCR templates.

DNA concentrations			
Sample dilutions	First biological replication	Second biological replication	Third biological replication
1/5 dilution	67.2 ng/µL	87.3 ng/µL	29.3 ng/µL
1/25 dilution	13.5 ng/µL	17.1 ng/µL	7 ng/µL
1/125 dilution	3.5 ng/µL	4.3 ng/µL	1.1 ng/µL
1/625 dilution	700 pg/µL	860 pg/µL	220 pg/µL
1/3125 dilution	140 pg/µL	172 pg/µL	44 pg/µL
1/15,625 dilution	28 pg/µL	34.4 pg/µL	8.8 pg/µL
1/78,125 dilution	5.6 pg/µL	6.88 pg/µL	1.8 pg/µL
1/390,625 dilution	1.1 pg/µL	1.4 pg/µL	352 fg/µL
1/1,953,125 dilution	224 fg/µL	275 fg/µL	70.4 fg/µL
1/9,765,625 dilution	44.8 fg/µL	55 fg/µL	14 fg/µL

### Preparation of viable *V. parahaemolyticus* cell PCR templates

The inoculum was prepared using overnight cultures of each ST36 strain grown in Alkaline Peptone Water (APW)^23^, broth at 35 °C with constant shaking at 180 rpm. Seed cultures were diluted 1/100 in 100 mL of freshly prepared APW broth and grown to an optical density at 600 nm of 0.4. After this, the cultures were centrifuged and washed three times then adjusted to 0.5 McFarland standard, which is comparable to a bacterial suspension of ~ 1.5 × 10^8^ colony-forming units (CFU) per 1 mL. After normalization of cultures, equal volumes of each ST36 strain culture were added and mixed to produce a final inoculum. This final inoculum was serially two-fold diluted, resulting in ten different cell dilutions. Cell numbers of inocula were determined by serial dilutions and spread plating on TSA supplemented with 2% of NaCl. Inocula cell numbers per mL and PCR reactions of each PCR template used in this experiment are provided in Table 3.

**Table 3:** Numbers of *Vibrio parahaemolyticus* cells for each inoculum and PCR template.

	First biological replication		Second biological replication		Third biological replication	
Sample dilutions	Cells per mL	Cells per PCR reaction	Cells per mL	Cells per PCR reaction	Cells per mL	Cells per PCR reaction
1/2	230,000	460	730,000	1460	400,000	800
1/4	115,000	230	365,000	730	200,000	400
1/8	57,500	115	182,500	365	100,000	200
1/16	28,750	57.5	91,250	182	50,000	100
1/32	14,375	29	45,625	91	25,000	50
1/64	7187	14	22,812	46	12,500	25
1/128	3594	7	11,406	23	6250	12.5
1/256	1797	3.5	5703	11	3125	6
1/512	898	1.8	2851	5.7	1562	3
1/1024	449	0.9	1425	2.8	781	1.5

### Spiking of mussel tissue

The inocula were prepared as described above. In total, seven different inocula dilutions were prepared using tenfold dilution steps. Cell numbers of inocula were determined as above and dilutions as well as cell numbers of inocula used for each biological replication are shown in Table 4**.** Once the inocula were prepared, 100 μL of *V. parahaemolyticus* suspension was added to 1 mL of homogenised raw mussel tissue (Table 4) followed by a brief mixing and incubation at room temperature for 1 h. After this brief incubation, 9 mL of APW broth was added to each spiked mussel sample followed by overnight incubation at 35 °C. After overnight incubation, 1 mL of suspension was taken and centrifuged at 10,000×*g* for 3 min. The supernatant was removed, and genomic DNA was extracted using a Qiagen DNeasy Tissue kit (Qiagen Inc., Valencia, CA) according to the manufacturer’s instructions. The concentration of DNA extracted from each spiked mussel sample is shown in Table 4.

**Table 4:** Cell numbers used for *Vibrio parahaemolyticus* inoculation of mussel tissues.

Fold dilutions	First biological replication		Second biological replication		Third biological replication	
	Inoculum/cells per mL	^a^Concentration of DNA (ng/µL)	Inoculum/cells per mL	^a^Concentration of DNA (ng/µL)	Inoculum/cells per mL	^a^Concentration of DNA (ng/µL)
10^−4^	15,600	122.6	6600	60.7	6300	191.8
10^−6^	156	67.9	66	126.3	63	86.2
10^−7^	16	80.1	6.6	116	6.3	85.8
10^−8^	1.6	49.1	0.66	36.8	0.63	22.8
10^−9^	0.16	27.6	0.066	41.9	0.063	27.4
10^−10^	0.016	35.3	0.0066	18.7	0.0063	29
10^−11^	0.0016	18	0.00066	16	0.00063	36.8

^a^Concentrations of DNA extracted from 1 mL of spiked and incubated mussel samples.

### Collection of mussels

Greenshell^™^ mussels (*Perna canaliculus*) were collected at an aquaculture farm in the Marlborough Sounds, New Zealand, over three summer months (January 2022, February and January 2023) and also over three winter months in 2022 (June, July and August). In total, 480 mussels were collected. Each month, 80 mussels were harvested and placed inside a polystyrene box with a coolant pad and delivered to The New Zealand Institute for Plant and Food Research Limited, Auckland, within 24 h.

### Processing of mussels, incubation, and DNA extraction

Immediately upon arrival, the exterior shell surface of each mussel was wiped using Tork Xpress paper hand towels then the weight and the total length of each mussel was recorded. The mussels were aseptically shucked and tissues of 13 mussels, including their intervalvular water, were homogenised using a pre-weighed sterile Waring blender cup, followed by seven decimal dilutions of the homogenised tissue in Phosphate Buffered Saline (PBS, pH 7.4). Each dilution was transferred into triplicate tubes of APW starting with 1 g of undiluted homogenate into 9 mL of APW. Suspensions of mussel tissues and APW were incubated for 24 h at 35 °C for enrichment. After the first 24 h, 1 mL of the culture from the first three dilutions of each series was subsampled into fresh 10 mL APW tubes and incubated for a further 24 h at 35 °C^24^. This was done to limit of the amount of mussel meat and other potential PCR reaction inhibitors in the least-diluted tubes. After incubation, 1 mL was taken from each turbid enrichment, followed by DNA extraction using the heat boiling method^25^. Briefly, 1 mL of enrichment suspension was centrifuged at 15,000×*g* for 10 min. The supernatant was removed, and the pellet was resuspended in 1 mL of molecular grade water followed by a second centrifugation at 15,000×*g* for 10 min. The resulting pellet was resuspended in 50 µL of molecular grade water, followed by boiling at 100 °C for 10 min. After the boiling, the samples were cooled on ice and stored at − 20 °C for further use.

### Statistical analysis

For the ddPCR assay, for each of the three types of samples (genomic DNA, *V. parahaemolyticus* cells, and mussel-tissue spiked with *V. parahaemolyticus*), a dilution level was identified which appeared to give signals just above the signal from the blank wells. The signals of three genes (*ureR*, *tlh* and *tdh*) from positive but diluted samples were compared with those from blank wells graphically and using Receiver Operating Characteristic (ROC) curves calculated using the R package pROC^26^. Limits of detection were calculated as the upper 95% confidence limit for signals from the blank cells, using a t-distribution.

## Results

### Sensitivity of the diagnostic assays to detect extracted and purified genomic DNA

**Figure 1:**
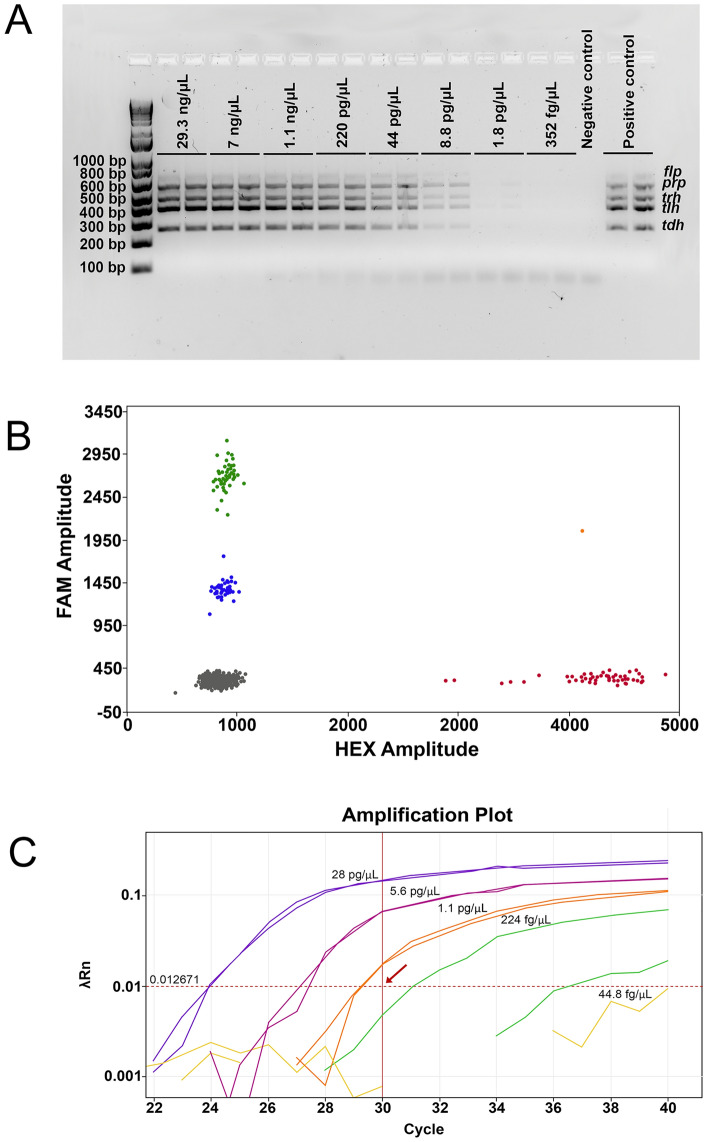
Sensitivity limits of PCR *Vibrio parahaemolyticus* assays as determined with serially diluted DNA. (**A**) Gel image shows the sensitivity limit of a multiplex ST36 PCR assay. Note, each DNA dilution was carried out in two technical replications. (**B**) ddPCR graph shows the presence and distribution of positive *tlh* (blue), *ureR* (green), *tdh* (red), *tdh* and *ureR* (orange) as well as negative (grey) droplets. The PCR reaction was carried out with 1.1 pg of genomic DNA. (**C**) Sensitivity of the qPCR assay using the last five dilution series (28 pg/µL, 5.6 pg/µL, 1.1 pg/µL, 224 fg/µL and 44.8 fg/µL) and targeting the *tdh* virulence marker. The *Ct* cut-off value (30) is indicated by the arrow. The qPCR was done in duplicate for each sample tested.

The sensitivities of the PCR platforms were determined using three biological and two technical replications with five-fold serially diluted DNA samples (Table 2). The results associated with biological replications where particular PCR assay achieved the lowest detection limit are presented. The multiplex ST36 PCR assay could detect all five genes, indicating the presence of non-pathogenic (*tlh*), pathogenic (*tdh*, *trh*) and ST36 (*prp*, *flp*) specific gene markers, in a sample containing 8.8 picograms (pg) of total DNA (Fig. 1A). This DNA sample was 1/15625 diluted compared with its original sample concentration and presented the lowest possible concentration of DNA that could be detected by the multiplex ST36 PCR assay (Fig. 1A). Using the same set of DNA samples, it was found that ddPCR and qPCR assays could detect the presence of virulence gene (*tdh*) in a sample containing 1.1 pg of DNA (1/390625 dilution) (Fig. 1B, C). However, the qPCR assay could not detect the presence of a non-pathogenic gene marker (*tlh*) in that sample (Fig. S1), whereas the ddPCR assay detected both the *tlh* gene (species-specific) and the *tdh* and *ureR* genes (pathogenic markers) (Fig. 1B), further indicating the presence of this non-pathogenic gene marker. To obtain reliable data indicating a limit of detection (LOD) for the ddPCR, the most sensitive assay, an additional six (third biological replication) and seven (first and second biological replications) technical replications, including negative controls, were carried out using a set of samples containing 1.1 pg of DNA (first biological replication), 1.4 pg of DNA (second biological replication) and 352 fg of DNA (third biological replication). This analysis showed that the copies of all three gene markers (*tlh*, *tdh* and *ureR*) for the biological replications one and two were significantly above those of negative controls (Fig. S2 and Table S1). In contrast, for the copies of the three genes associated with the biological replication three were not distinguishable from those of the negative controls (Fig. S2 and Table S1). In other words, the ddPCR could detect all three genomic markers in a sample containing 1.1 pg of DNA, whereas the detection signals were lost in a sample containing 0.3 pg (352 fg) of DNA.

In summary, the ddPCR and qPCR assays showed a similar level of sensitivity in the detecting genomic DNA of *V. parahaemolyticus*, whereas the conventional ST36 PCR assay exhibited a lower sensitivity.

### Detection of viable *V. parahaemolyticus* cells

**Figure 2:**
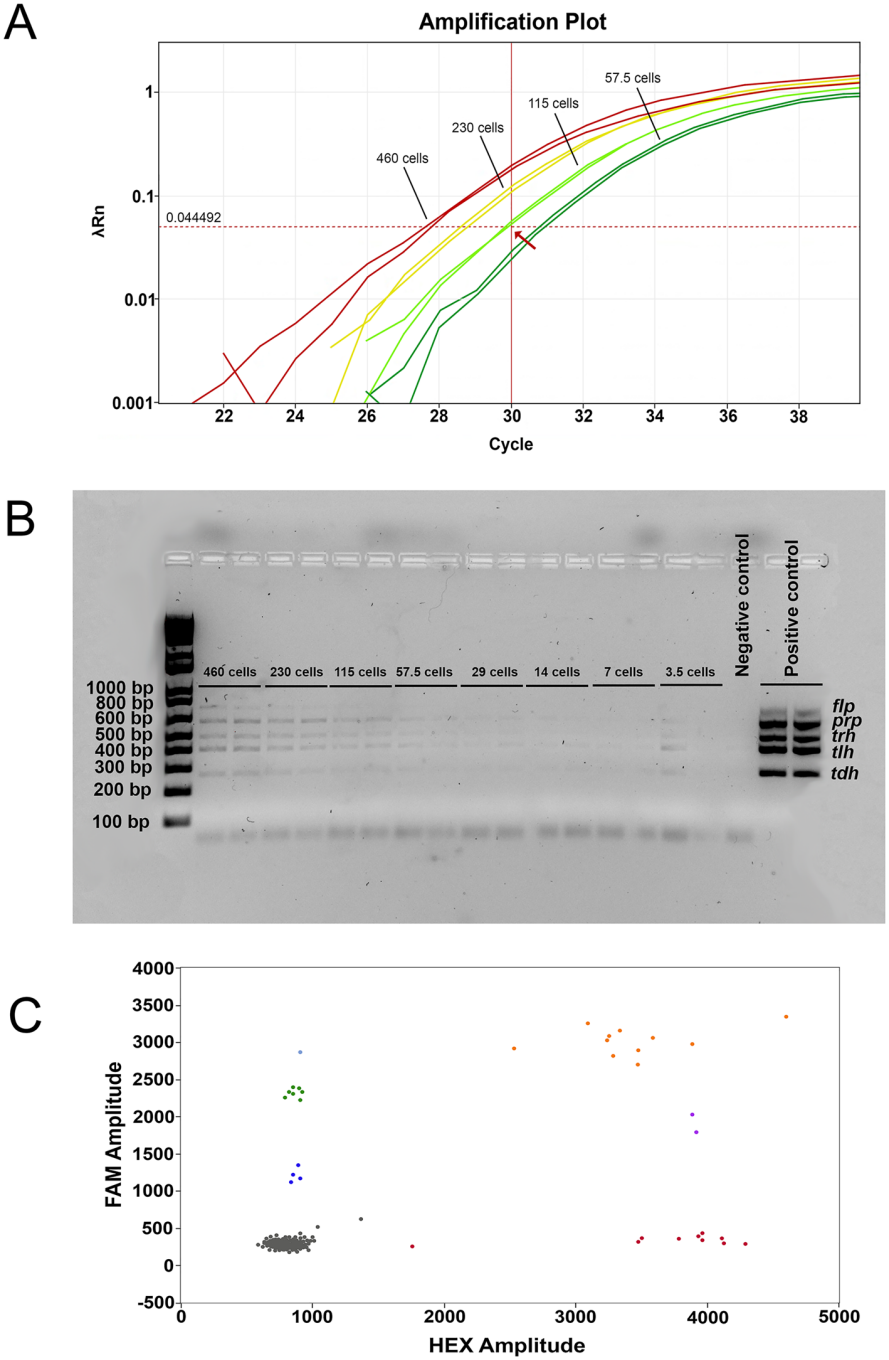
Sensitivity limits of PCR assays as determined with viable *Vibrio parahaemolyticus* cells (colony-PCR approach). (**A**) Amplification plot showing the limit of detection for the qPCR assay. The graph shows the results for four different samples each containing 460 cells, 230 cells, 115 cells and 57.5 cells, respectively. The red arrow indicates the cut-off value for this PCR assay. (**B**) Gel image portraying the limit of detection for the multiplex ST36 PCR assay. (**C**) ddPCR graph showing the presence and distribution of the positive *tlh* (dark blue), *ureR* (green), *tdh* (red), *tlh* and *ureR* (light blue), *tdh* and *ureR* (purple), *tlh*, *tdh* and *ureR* (orange) and negative (grey) droplets. The template contained 29 cells of *V. parahaemolyticus*.

Using viable cells of *V. parahaemolyticus* as a PCR template (a colony-PCR approach), the qPCR assay was able to detect the presence of non-pathogenic (*tlh*) and pathogenic (*tdh*) markers in a sample containing 115 cells (Fig. 2A). The conventional ST36 PCR assay could detect the presence of this food-borne pathogen in a sample containing 57 cells (Fig. 2B), a better level of sensitivity than the qPCR assay. Testing the sensitivity of the ddPCR assay on the same set of samples, it was found that this PCR assay could detect the presence of pathogenic (*tdh* and *ureR*) and non-pathogenic (*tlh*) markers in a sample containing 29 cells (Fig. 2C). To increase the robustness of the data for the ddPCR, an additional seven (third biological replication) and eight (first and second biological replications) technical replications of each biological replication were tested, including samples that contained 29 cells (first biological replication), 91 cells (second biological replication) and 50 cells (third biological replication). This additional analysis showed that the ddPCR assay could detect 0.7 copies of *ureR*, 1.09 copies of *tlh* and 0.93 copies of *tdh* per 1 µL of the sample that contained 29 cells in total, which was significantly above the detection in the negative control samples (Fig. S3 and Table S2). The colony-PCR approach demonstrated that the ddPCR was the most sensitive PCR platform in detecting viable cells of *V. parahaemolyticus*.

### Detection of *V. parahaemolyticus* in spiked mussel samples

**Figure 3:**
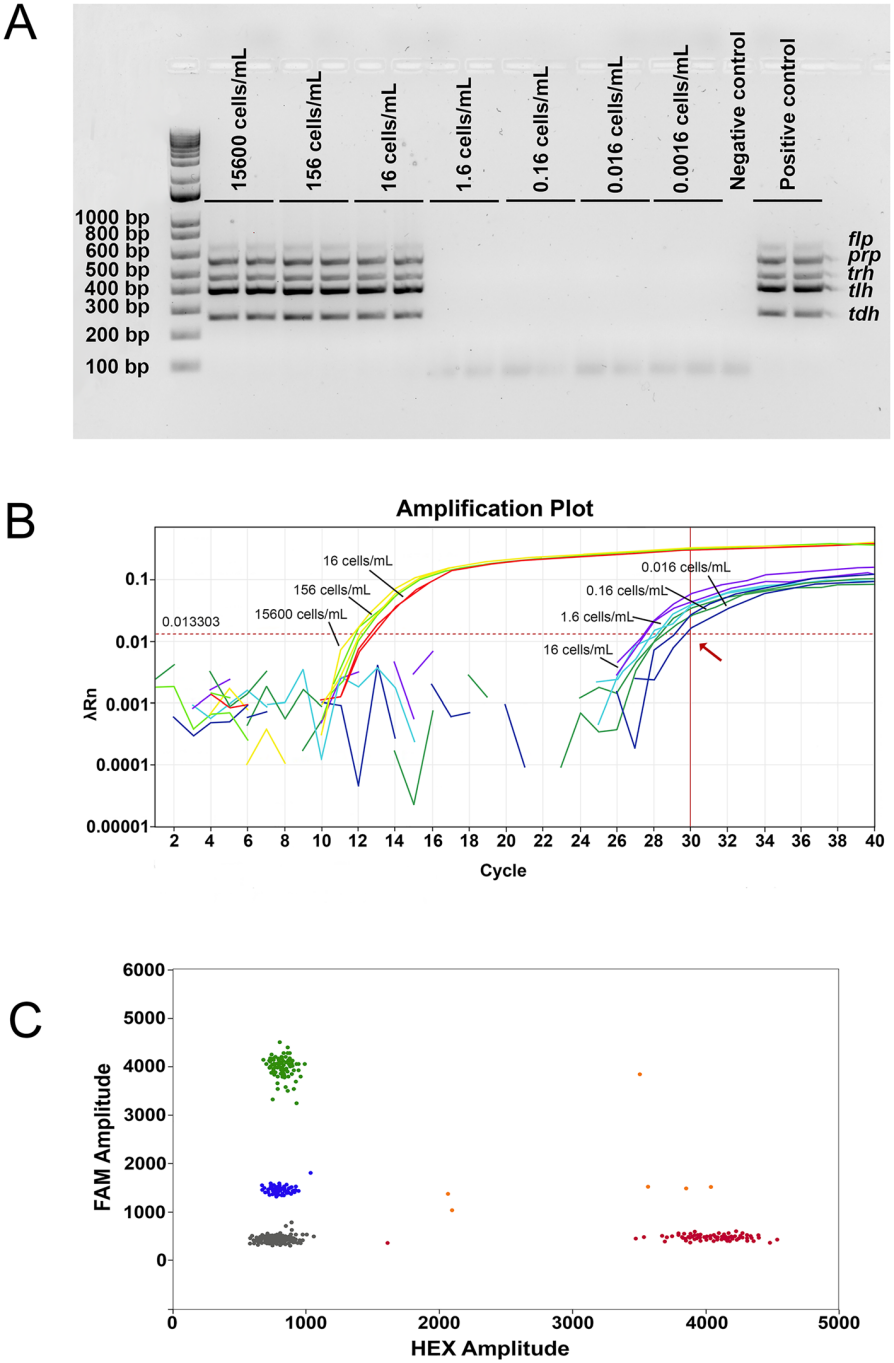
Sensitivity limits of PCR assays with mussel tissues spiked with *Vibrio parahaemolyticus*. (**A**) Gel image portraying the limit of detection for the multiplex ST36 PCR assay. (**B**) Amplification graph showing the limit of detection for the qPCR assay. The graph shows the results for all samples, grouping them into two groups: (i) strong positive (first three dilution series), and (ii) weak positive (the remaining four dilution series). The red arrow indicates the cut-off value for this PCR assay. (**C**) ddPCR graph showing the presence and distribution of the positive *tlh* (dark blue), *ureR* (green), *tdh* (red), *tdh* and *tlh* (purple) and *tlh*, *tdh* and *ureR* (orange) and negative (grey) droplets.

After conducting the tests using three biological replications of spiked mussel tissue, the conventional ST36 PCR assay detected all five genetic markers (*tdh*, *tlh*, *trh*, *prp* and *flp*) in the samples inoculated with 10^−7^ inoculum (Table 4) or 16 cells (Fig. 3A). After this, no diagnostic bands could be detected in the samples inoculated with more diluted inocula (Fig. 3A), indicating that the 10^−7^ inoculum was the LOD for the conventional ST36 PCR assay. In the case of the qPCR assay, a strong detection signal (10–15 Ct) was observed in the samples inoculated with the first three inocula (10^−4^, 10^−6^ and 10^−7^), whereas in the rest of the samples, inoculated with more diluted inocula (10^−8^, 10^−9^, 10^−10^ and 10^−11^), was observed a weak positive diagnostic signal (25–30 Ct) (Fig. 3B). Using the identical set of samples, the ddPCR assay also showed the presence of *V. parahaemolyticus* in all samples. The diagnostic signal for the samples inoculated with the most diluted inoculum (10^–11^) for all three biological replications was in the ranges of 2.71 to 5.4 copies per µL for the *ureR* gene, 4.37 to 10.3 copies per µL for the *tlh* gene, and 2.91 to 6.31 copies for the *tdh* gene (Fig. 3C). Conducting an additional ddPCR analysis that included eight technical replications for each biological replication of the samples inoculated with the most diluted inoculum, showed that the mean value ranged for the *ureR* gene from 1.52 to 5.82 copies, for the *tlh* gene between 3.18 and 12.25 copies, and for the *tdh* gene the mean value ranged from 1.78 to 6.40 copies per 1 µL (Fig. S4 and Table S3). These mean values were significantly above those of the negative control values (from 0.10 to 0.13 copies), clearly indicating the presence of *V. parahaemolyticus*. The experiment with the spiked mussel tissue showed that the conventional ST36 PCR assay was the least sensitive, whereas the qPCR and ddPCR assays showed greater sensitivities.

### Ability of the diagnostic assays to detect *V. parahaemolyticus* in naturally contaminated mussels

**Figure 4:**
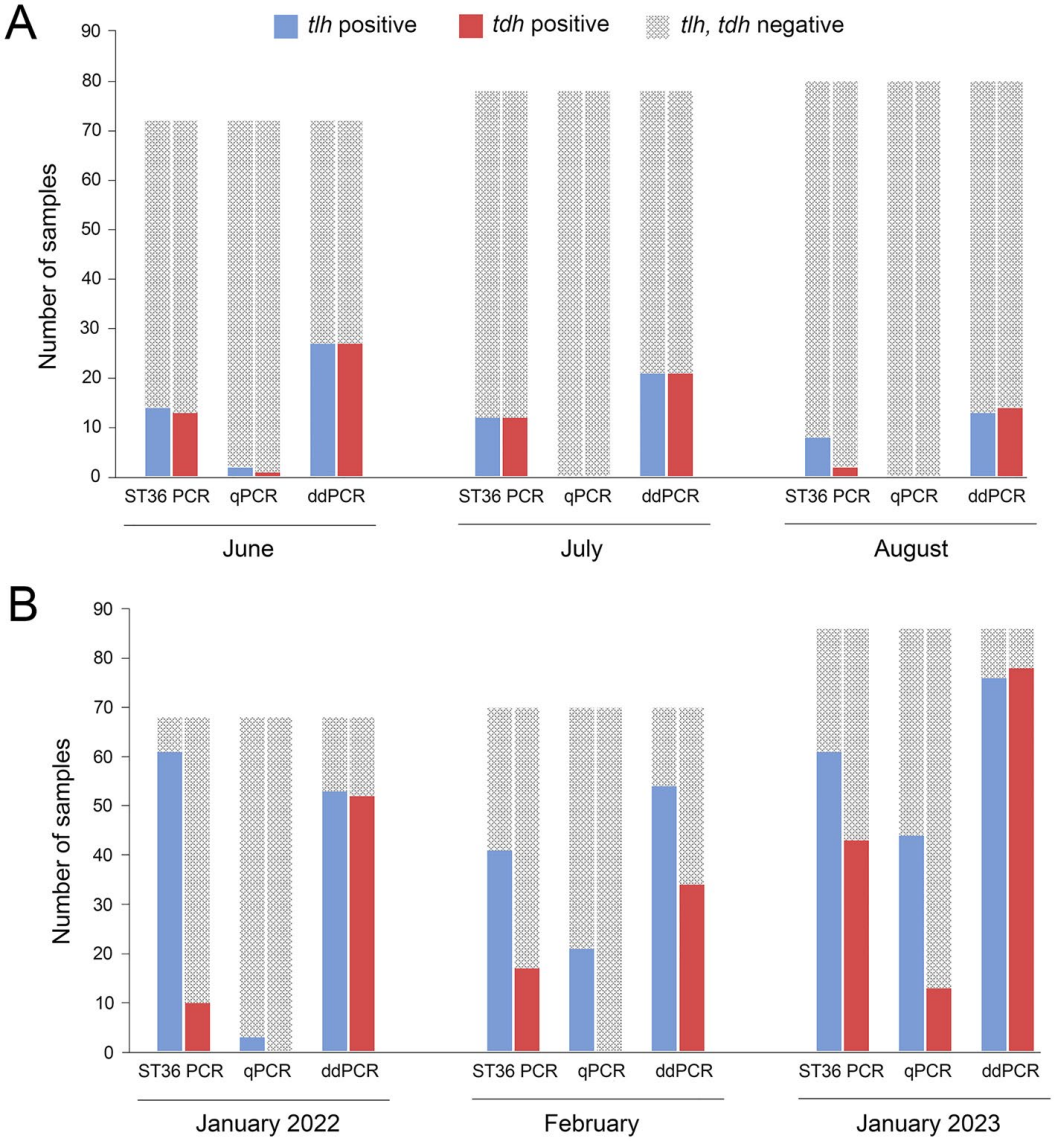
Sensitivities of conventional ST36 PCR, qPCR and ddPCR in the detection of *Vibrio parahaemolyticus* from naturally contaminated mussels. (**A**) In winter months, mussels were harvested in seawater that ranged from 11.8 °C (July) and 12.2 °C (August) to 14.6 °C (June). (**B**) During summer months, mussels were harvested in seawater that ranged from 18.7 °C (January) to 19.5 °C (February).

The naturally contaminated mussels were harvested during two distinct temperature periods in the Southern Hemisphere, in winter months (June–July, and August) and summer months (January 2022, February, and January 2023). In general, during the winter months, the prevalence of *V. parahaemolyticus* in mussels was significantly lower than in the summer months (Fig. 4A, B). Bacterial DNA from all mussel samples was extracted by the boiling method. For the mussels harvested in June, the ddPCR assay detected 37.5% (*n* = 27) positive samples for *V. parahaemolyticus* (*tlh*), while the conventional ST36 PCR assay detected 19.4% (*n* = 14) and qPCR showed 2.7% (*n* = 2) of samples positive for *V*. *parahaemolyticus* (Fig. 4A). Similarly, the ddPCR assay detected 37.5% (*n* = 27) of samples being positive for the virulence factor, *tdh*, whereas the conventional ST36 PCR indicated 18% (*n* = 13) and the qPCR showed 1.3% (*n* = 1) of samples being positive for the same virulence marker (Fig. 4A). For the samples harvested in July, the ddPCR assay detected 26.9% (*n* = 21) of samples being positive for the presence of *V. parahaemolyticus* (*tlh*) and its virulence marker (*tdh*), while the conventional ST36 PCR showed 15.4% (*n* = 12) of samples positive for both *tlh* and *tdh* (Fig. 4A). The qPCR assay detected neither *tlh* nor *tdh* in the same set of samples. For the last winter month, August, the ddPCR identified 16.2% (*n* = 13) *tlh*-positive and 17% (n = 14) *tdh*-positive samples, whereas the conventional ST36 PCR showed 10% (*n* = 8) positive samples for *tlh* and 2.5% (*n* = 2) positive for *tdh* (Fig. 4A). The qPCR could detect neither *tlh* nor *tdh* in mussels harvested during August (Fig. 4A). For the first time, testing the mussels harvested in January 2022, the conventional ST36 PCR assay showed a greater sensitivity, detecting 89.7% (*n* = 61) of samples positive for *V. parahaemolyticus* compared with 77.9% (*n* = 53) positive samples identified by the ddPCR assay (Fig. 4B). The conventional ST36 PCR assay detected 14.7% (*n* = 10) of samples with the *tdh* virulence marker, whereas the ddPCR assay showed 76.4% (*n* = 52) of samples being positive for the *tdh* gene (Fig. 4B). The qPCR assay detected 4.4% (*n* = 3) of samples as positive for *V. parahaemolyticus* and no samples positive for the *tdh* virulence factor (Fig. 4B). For the mussels harvested in February, the ddPCR assay detected 77.1% (*n* = 54) of samples positive for the presence of *V. parahaemolyticus* and 48.5% (*n* = 34) of samples positive for the *tdh* gene (Fig. 4B). At the same time, the conventional ST36 assay detected 58.6% (*n* = 41) of samples positive for *V. parahaemolyticus* and 24.3% (*n* = 17) of samples positive for the *tdh* virulence factor (Fig. 4B). The qPCR assay identified 30% (*n* = 21) samples positive for *V. parahaemolyticus* and did not detect any presence of the *tdh* gene (Fig. 4B). For the mussels harvested during January 2023, the ddPCR assay detected 88.3% (*n* = 76) of samples as positive for *V. parahaemolyticus*, followed by the conventional ST36 PCR assay, 71% (*n* = 61) and the qPCR assay, 51.1% (*n* = 44) (Fig. 4B). A similar trend was observed in the detection of the virulence factor, *tdh*, with the ddPCR assay being the most sensitive, resulting in 90.7% (n = 78) of positive samples, followed by the conventional ST36 PCR with 50% (*n* = 43), and the qPCR assay with 15.1% (*n* = 13) positive samples, respectively (Fig. 4B).

## Discussion

To reveal the strengths and weaknesses of different diagnostic methods employed for the detection of *V. parahaemolyticus,* it is important to evaluate their sensitivities using various testing approaches. In this study, the sensitivities of three PCR assays, based on three platforms, (*i*) conventional PCR, (*ii*) qPCR and (*iii*) ddPCR, were determined against sets of samples containing purified genomic DNA, viable cells, spiked mussel tissue, and naturally contaminated mussels. Also, two sample preparation methods for DNA extraction were used, the Qiagen DNeasy Tissue kit and the heat boiling method.

Using purified genomic DNA derived from *V. parahaemolyticus* and testing the LOD of the employed PCR assays, it was found that the ddPCR exhibited the greatest LOD, detecting the presence of *V. parahaemolyticus* (*tlh*) and its virulence markers (*tdh* and *ureR*) in a sample containing 1.1 pg/µL of highly purified DNA. The qPCR assay was capable of detecting *V. parahaemolyticus* in the same sample but failed to detect the targeted pathogenic marker (*tdh*). Zhu and colleagues^27^, using the real-time recombinase polymerase amplification PCR approach for the detection of the *tlh* gene, reported 0.4 pg/µL of DNA as the LOD for this assay. Another group of authors, employing a multiplex qPCR with TaqMan fluorescent probes, reported that 200 pg of purified genomic DNA was the LOD for this PCR assay^28^. Note that the real-time recombinase polymerase amplification assay targeted only a single genetic marker (*tlh*), whereas the qPCR with TaqMan fluorescent probes and the PCR assays used in this study targeted multiple genetic markers simultaneously. It has been reported that the use of the multiplex PCR approach causes competition between the amplifications of multiple genes, resulting in significant decreases of LOD for the employed multiplex PCR assays^29^. If one gene is significantly more abundant compared to other genes in a multiplex reaction, this may lead to reaching its plateau phases much earlier. This usually results in the limitation of nucleotides and other reagents for the remaining genes, causing their limited amplification. The phenomenon of the competition between amplifications of multiple genes in the multiplex PCR format may explain why the qPCR assay employed in our study was able to detect the *tdh* gene but could not detect the presence of the *tlh* gene in a sample that contained the same number of the *tdh* and *tlh* gene copies.

The colony-PCR approach revealed that the ddPCR exhibited the greatest LOD, compared with those of the conventional PCR and qPCR assays. Interestingly, the conventional PCR assay showed greater sensitivity than the qPCR assay in detecting the viable *V. parahaemolyticus* cells, whereas the qPCR assay was more sensitive than the conventional PCR in the detection of *V. parahaemolyticus* via the purified genomic DNA. These results indicate that the type of genomic template can play a role in the overall sensitivity of different PCR assays in the detection of *V. parahaemolyticus*. Indeed, Jones et al. (2012) observed a significant difference in the sensitivity of the real-time PCR assay^29^ in detecting *V. parahaemolyticus* when it was run using the BAX^®^ System lysis (*p* = 0.0004) for the PCR template preparation versus the heat boiling method (*p* = 0.0347)*.*

Testing the spiked mussel tissue samples, great difference was observed between the conventional PCR assay on one hand and the ddPCR and qPCR assays on the other hand. While the conventional PCR assay was able to detect the presence of *V. parahaemolyticus* and its pathogenic markers only in the mussel samples inoculated with the first three inocula, the ddPCR and qPCR identified this organism together with the *tdh* gene in the samples inoculated with all seven inocula. The literature commonly reports that food matrices, especially shellfish, contain a wide range of potent PCR inhibitors that can potentially lead to the generation of false-negative results^30–32^^.^ The presence of PCR inhibitors is unlikely to have affected the conventional PCR assay as the spiked mussel tissue samples underwent the enrichment step followed by the DNA extraction and purification, further ensuring a high quality of PCR templates for this experimental approach. The most likely explanation for the weak sensitivity of the conventional PCR assay compared with that of the qPCR assay lies in the fact that the qPCR shows a greater sensitivity with purified DNA, as already shown in the experiment with serially diluted genomic DNA.

Finally, the sensitivities of the PCR assays were determined against a large number of naturally contaminated mussels that were harvested during the winter and summer months in a region known to produce low concentrations of *V. parahaemolyticus* in mussels. A rationale for this experimental approach was to test the PCR assays against mussels that were naturally contaminated with low (winter) and higher (summer) numbers of *V. parahaemolyticus* cells. Overall, the ddPCR assay showed superiority over the other two PCR platforms in detecting not only the non-pathogenic marker of *V. parahaemolyticus* but also the pathogenic markers over the winter and summer months. Only once (testing mussels that were harvested in January 2022) was the conventional PCR assay capable of exhibiting a slightly greater sensitivity than the ddPCR assay in detecting the presence of *V. parahaemolyticus* (*tlh*). The initial testing of the mussels, harvested during the summer months, suffered an inability of the ddPCR assay to generate separate clusters of positive droplets, rather generating a single cluster positioned at the central point of the graph. This failure was caused by the high abundance of genetic material, causing mispriming and/or loss of amplification. It has been reported that a high abundance of PCR templates can lead to amplification failure or mispriming, resulting in overall PCR reaction failure^33^. Indeed, a dilution of these samples led to the generation of distinct clusters with positive droplets, allowing absolute quantification of the targeted gene loci. Regarding the other two PCRs platforms, the conventional PCR assay significantly outperformed the qPCR assay in detecting the total and pathogenic populations of *V. parahaemolyticus* in naturally contaminated mussels over the entire course of the study. It is important to mention that the detection of *V. parahaemolyticus* from the naturally contaminated mussels was carried out using the heat boiling method. This DNA extraction method does not purify genomic material and therefore a wide range of PCR inhibitors can be present in these samples. From the literature is known that ddPCR exhibits high resilience to various PCR inhibitors derived from plants, soil and wastewater compared to other PCR platforms^34^. The greater sensitivity of the ddPCR in detecting *V. parahaemolyticus* from PCR inhibition prone samples can be explained by the high resilience of ddPCR to various PCR inhibitors.

In summary, the sensitivities of three PCR assays, based on three PCR platforms, were systematically compared using laboratory and environmentally based approaches. The ddPCR assay demonstrated consistently great sensitivity in detecting *V. parahaemolyticus* in various types of samples, including PCR inhibition prone samples. In contrast to the ddPCR assay, the conventional PCR and qPCR assays showed significant fluctuations in their sensitivities, depending on the type of tested samples. In general, the conventional PCR assay showed significantly greater sensitivity than that of the qPCR assay in detecting *V. parahaemolyticus* in crude samples (naturally contaminated mussels and viable cells), whereas the qPCR assay showed a better sensitivity than the conventional PCR in detecting the presence of *V. parahaemolyticus* in purified DNA samples (purified genomic DNA and spiked mussels).

### Supplementary Information

Supplementary Figure S1.

Supplementary Figure S2.

Supplementary Figure S3.

Supplementary Figure S4.

Supplementary Information.

## Data Availability

Requests for access to the data for research purposes can be sent to Sinisa.Vidovic@plantandfood.co.nz.
