# Supplementary figures and images for: Detection of non-pathogenic and pathogenic populations of Vibrio parahaemolyticus in various samples by the conventional, quantitative and droplet digital PCRs
Source: Sci Rep. 2024 Feb 19;14:4137. doi: 10.1038/s41598-024-54753-y (PMC10876695; doi:10.1038/s41598-024-54753-y)

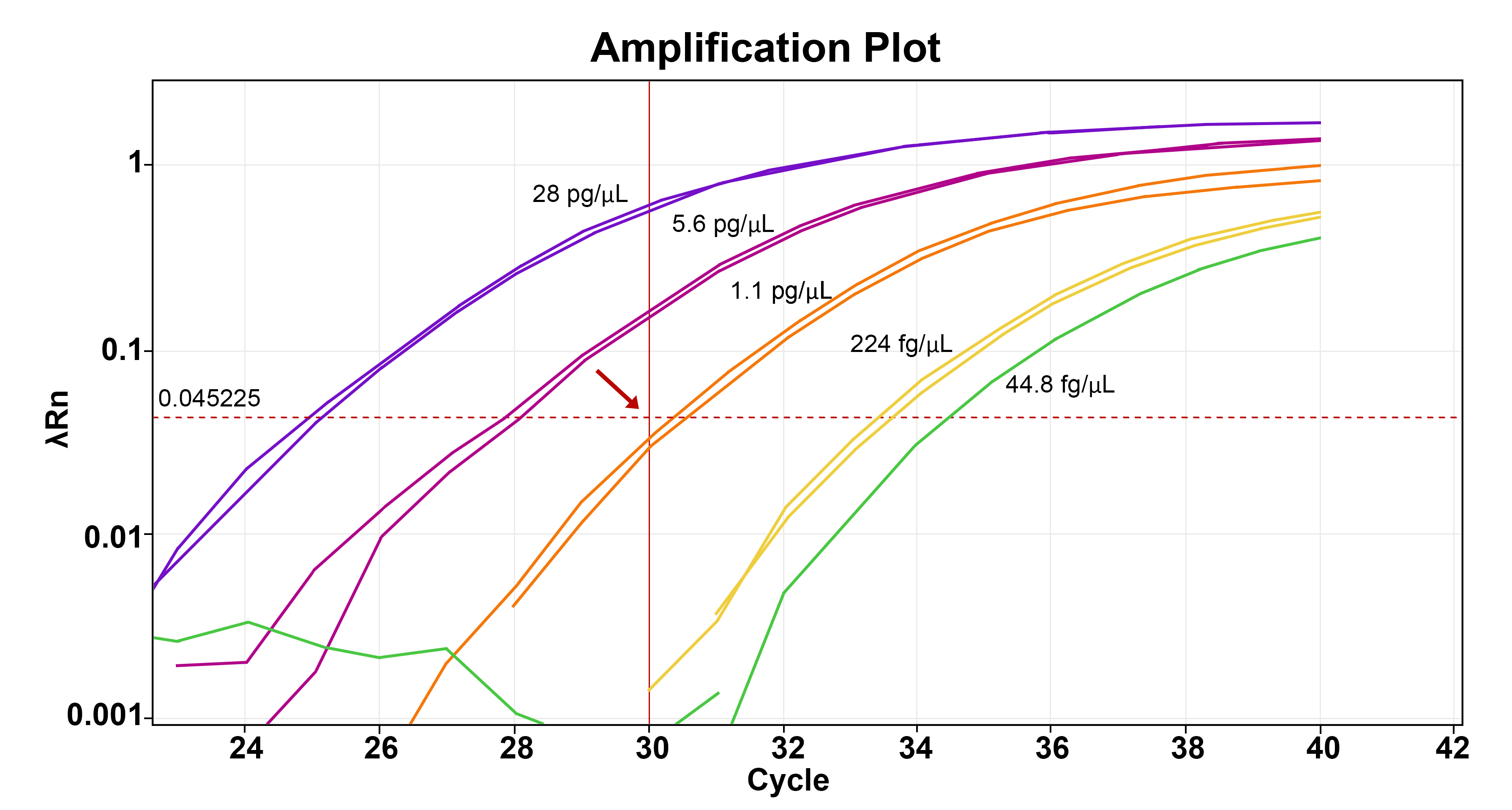

Supplement: Supplementary file 1 — Supplementary Figure S1. [file 41598_2024_54753_MOESM1_ESM.tif]

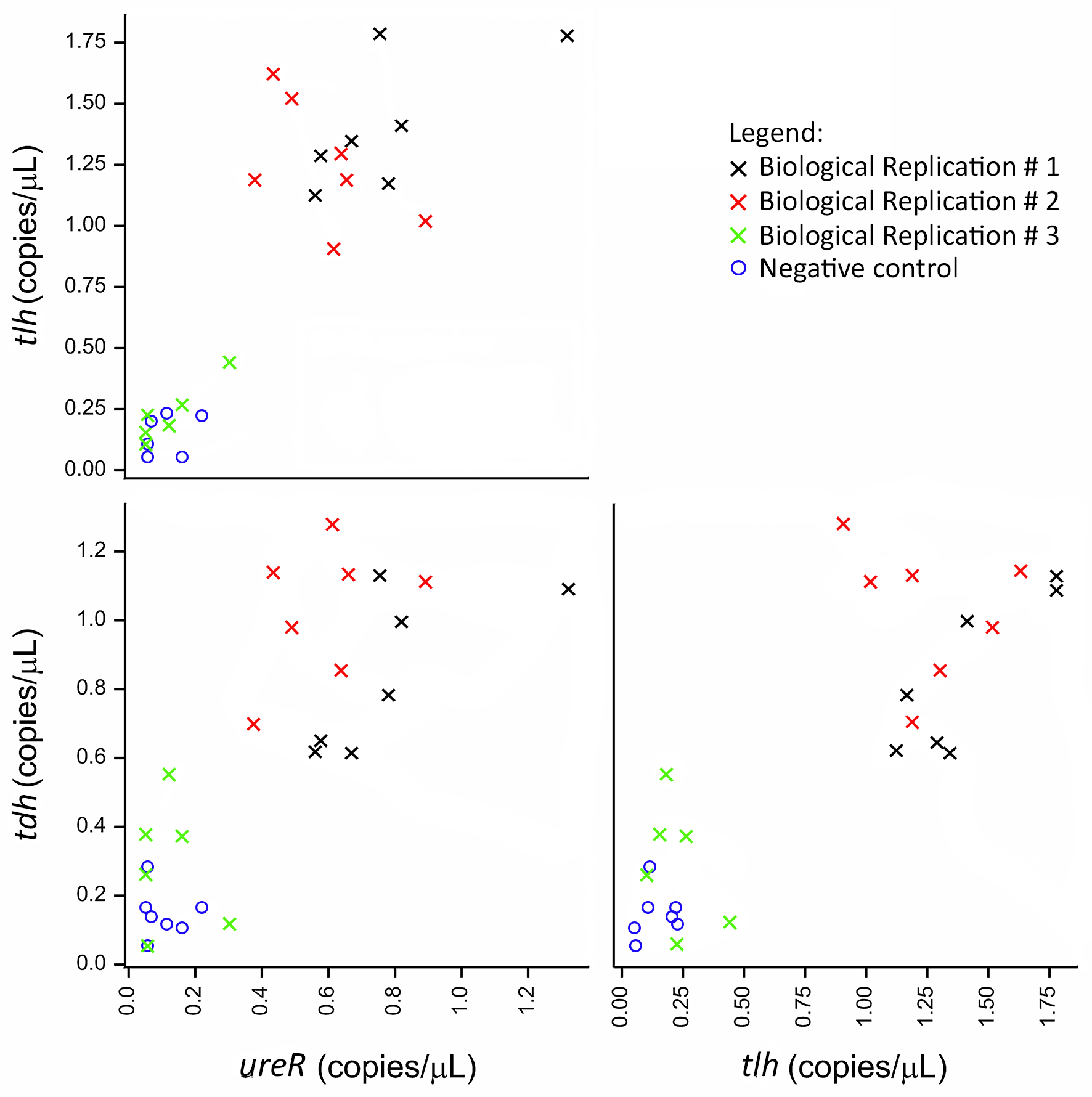

Supplement: Supplementary file 2 — Supplementary Figure S2. [file 41598_2024_54753_MOESM2_ESM.tif]

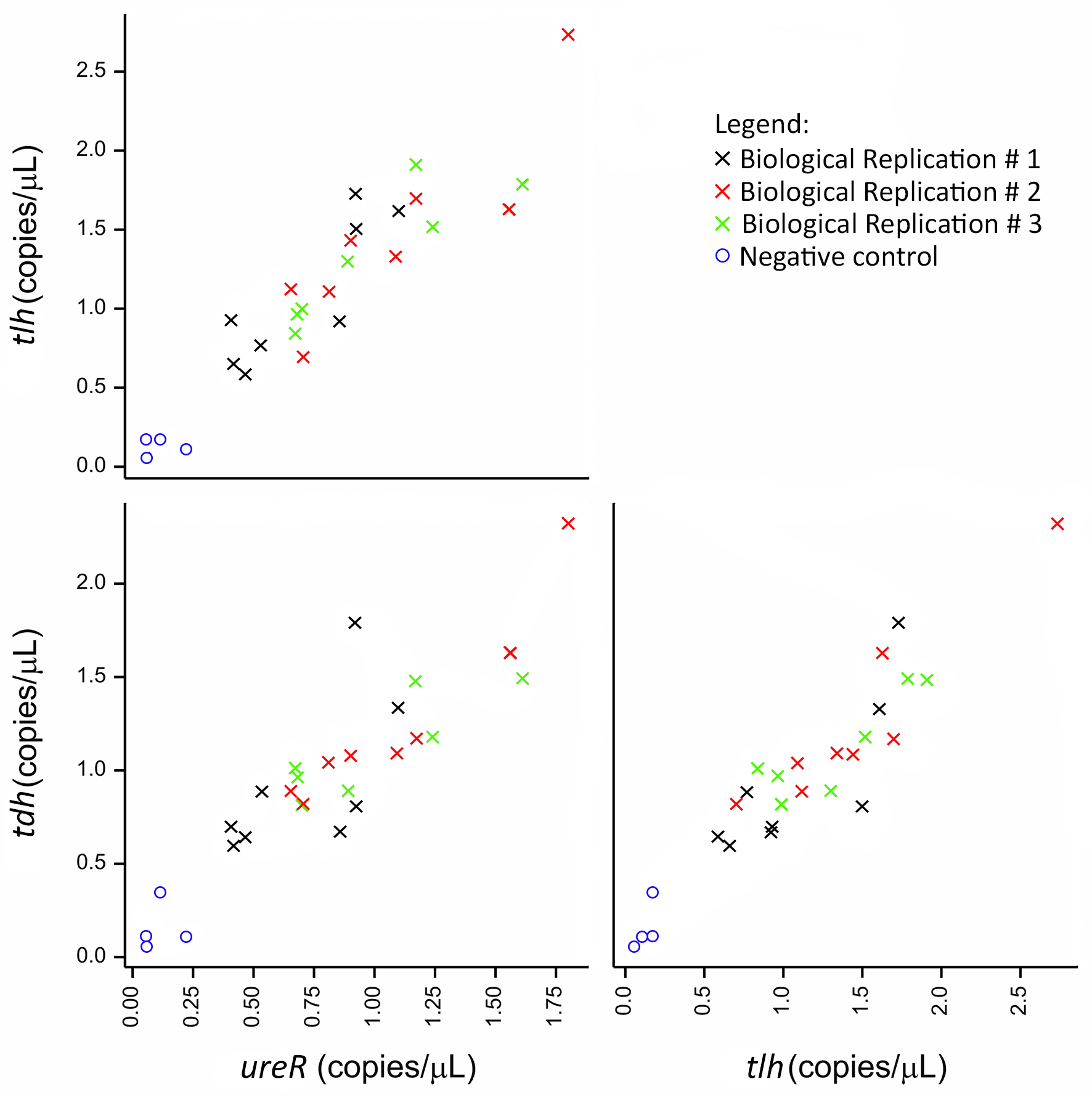

Supplement: Supplementary file 3 — Supplementary Figure S3. [file 41598_2024_54753_MOESM3_ESM.tif]

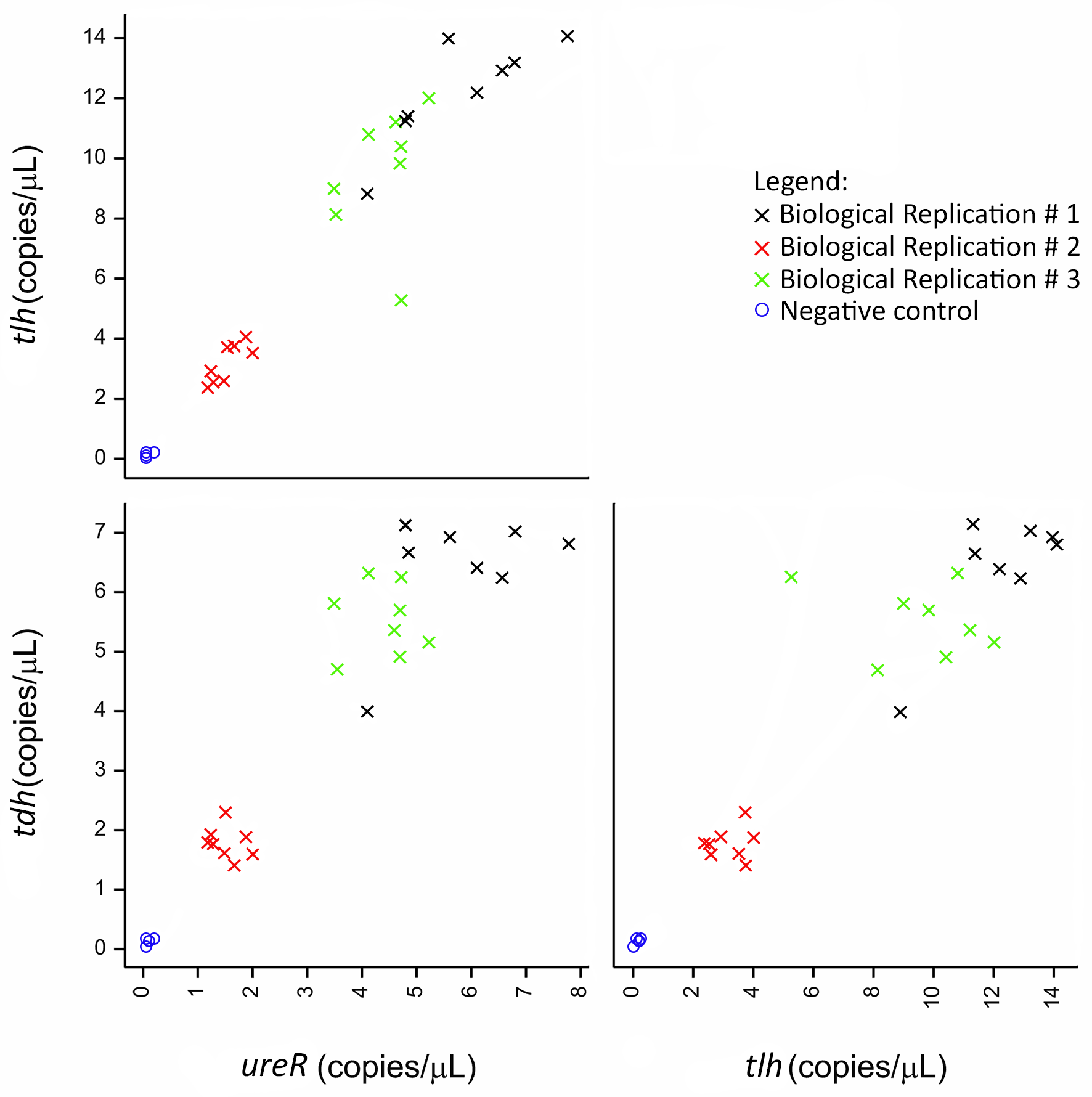

Supplement: Supplementary file 4 — Supplementary Figure S4. [file 41598_2024_54753_MOESM4_ESM.tif]
