# Supplementary material for: Detection of non-pathogenic and pathogenic populations of Vibrio parahaemolyticus in various samples by the conventional, quantitative and droplet digital PCRs
Source: Sci Rep. 2024 Feb 19;14:4137. doi: 10.1038/s41598-024-54753-y (PMC10876695; doi:10.1038/s41598-024-54753-y)
Supplement: Supplementary file 5 — Supplementary Information. [file 41598_2024_54753_MOESM5_ESM.docx]

**Supplementary materials**

**Figure S1.** Purified *Vibrio* *parahaemolyticus* DNA. An amplification shows the sensitivity of the qPCR assay using the last five dilution series (28 pg/µL, 5.6 pg/µL, 1.1 pg/µL, 224 fg/µL and 44.8 fg/µL) and targeting the *tlh* species-specific marker. The *Ct* cut-off value (30) is indicated by the arrow.

**Figure S2.** Purified *Vibrio* *parahaemolyticus* DNA. Additional seven technical replications were carried out by the ddPCR assay for all three biological replications. The PCR templates contained 1.1.pg/µL, 1.4 pg/µL and 352 fg/µL of DNA for the first, second and third biological replications, respectively.

**Figure S3.** Viable *Vibrio* *parahaemolyticus* cells. Additional eight technical replications for the first two biological replications and seven technical replications for the third biological replications were carried out by the ddPCR assay. The PCR templates contained 29, 91 and 50 cells for the first, second and third biological replications, respectively.

**Figure S4.** Mussel tissue spiked with *Vibrio parahaemolyticus*. Additional eight technical replications were carried out by the ddPCR assay for all three biological replications. The PCR templates contained 18 ng/µL, 16 ng/µL, and 36.8 ng/µL for the first, second and third biological replications, respectively.

**Table S1.** Genomic *Vibrio* *parahaemolyticus* DNA. Mean values for diagnostic markers of ddPCR.

|  |  |  | Diagnostic markers | | | | | | | |
| --- | --- | --- | --- | --- | --- | --- | --- | --- | --- | --- |
|  |  |  | *ureR* | |  | *tlh* | |  | *tdh* | |
| Type of sample | Number of tech. replications |  | Mean | Standard dev. |  | Mean | Standard dev. |  | Mean | Standard dev. |
| Negative control | 7 |  | 0.10 | 0.06 |  | 0.14 | 0.08 |  | 0.15 | 0.07 |
| Biological replication I | 7 |  | 0.78 | 0.26 |  | 1.41 | 0.27 |  | 0.84 | 0.23 |
| Biological replication II | 7 |  | 0.59 | 0.17 |  | 1.25 | 0.26 |  | 1.03 | 0.20 |
| Biological replication III | 6 |  | 0.12 | 0.10 |  | 0.23 | 0.12 |  | 0.29 | 0.18 |

**Table S2.** *Vibrio* *parahaemolyticus* cell culture. Mean values for diagnostic markers of ddPCR.

|  |  |  | Diagnostic markers | | | | | | | |
| --- | --- | --- | --- | --- | --- | --- | --- | --- | --- | --- |
|  |  |  | *ureR* | |  | *tlh* | |  | *tdh* | |
| Type of sample | Number of tech. replications |  | Mean | Standard dev. |  | Mean | Standard dev. |  | Mean | Standard dev. |
| Negative control | 4 |  | 0.11 | 0.08 |  | 0.13 | 0.05 |  | 0.16 | 0.13 |
| Biological replication I | 8 |  | 0.70 | 0.28 |  | 1.09 | 0.46 |  | 0.93 | 0.42 |
| Biological replication II | 8 |  | 1.09 | 0.41 |  | 1.47 | 0.60 |  | 1.26 | 0.49 |
| Biological replication III | 7 |  | 0.99 | 0.36 |  | 1.33 | 0.42 |  | 1.12 | 0.27 |

**Table S3.** Mussels spiked with *Vibrio* *parahaemolyticus*. Mean values for diagnostic markers of ddPCR.

|  |  | Diagnostic markers | | | | | |
| --- | --- | --- | --- | --- | --- | --- | --- |
|  |  | *ureR* |  | *tlh* |  | *tdh* |  |
| Type of sample | Number of tech. replications | Mean | Standard dev. | Mean | Standard dev. | Mean | Standard dev. |
| Negative control | 4 | 0.10 | 0.08 | 0.15 | 0.08 | 0.13 | 0.05 |
| Biological replication I | 8 | 5.82 | 1.22 | 12.25 | 1.72 | 6.40 | 1.02 |
| Biological replication II | 8 | 1.52 | 0.30 | 3.18 | 0.65 | 1.78 | 0.26 |
| Biological replication III | 8 | 4.38 | 0.62 | 9.58 | 2.14 | 5.53 | 0.60 |
